# Dr. Henfrey

**Published:** 1860-01

**Authors:** 


DR. HENFREY, the learned Professor of Botany in King's College, London, died on the seventh of September, in the thirty-ninth year of his age. He was the author of several works on the vegetable kingdom.

